# Deviance-Related Responses along the Auditory Hierarchy: Combined FFR, MLR and MMN Evidence

**DOI:** 10.1371/journal.pone.0136794

**Published:** 2015-09-08

**Authors:** Tetsuya Shiga, Heike Althen, Miriam Cornella, Katarzyna Zarnowiec, Hirooki Yabe, Carles Escera

**Affiliations:** 1 Institute for Brain, Cognition and Behavior (IR3C), University of Barcelona, Barcelona, Catalonia, Spain; 2 Cognitive Neuroscience Research Group, Department of Psychiatry and Clinical Psychobiology, University of Barcelona, Barcelona, Catalonia, Spain; 3 Department of Neuropsychiatry, Fukushima Medical University, Fukushima, Fukushima, Japan; University of Salamanca- Institute for Neuroscience of Castille and Leon and Medical School, SPAIN

## Abstract

The mismatch negativity (MMN) provides a correlate of automatic auditory discrimination in human auditory cortex that is elicited in response to violation of any acoustic regularity. Recently, deviance-related responses were found at much earlier cortical processing stages as reflected by the middle latency response (MLR) of the auditory evoked potential, and even at the level of the auditory brainstem as reflected by the frequency following response (FFR). However, no study has reported deviance-related responses in the FFR, MLR and long latency response (LLR) concurrently in a single recording protocol. Amplitude-modulated (AM) sounds were presented to healthy human participants in a frequency oddball paradigm to investigate deviance-related responses along the auditory hierarchy in the ranges of FFR, MLR and LLR. AM frequency deviants modulated the FFR, the Na and Nb components of the MLR, and the LLR eliciting the MMN. These findings demonstrate that it is possible to elicit deviance-related responses at three different levels (FFR, MLR and LLR) in one single recording protocol, highlight the involvement of the whole auditory hierarchy in deviance detection and have implications for cognitive and clinical auditory neuroscience. Moreover, the present protocol provides a new research tool into clinical neuroscience so that the functional integrity of the auditory novelty system can now be tested as a whole in a range of clinical populations where the MMN was previously shown to be defective.

## Introduction

Humans have evolved the ability to attend to potentially relevant novel events even when occurring outside the current focus of attention. A large body of evidence suggests that our auditory system compares incoming sounds with sensory memory traces derived from ongoing regularities [1] and triggers an error signal in order to allocate appropriate processing resources to unexpected changes in the acoustic environment [2]. A well-characterized component of the auditory evoked potential (AEP), the so-called mismatch negativity (MMN) [3], has been identified as the neural correlate of auditory deviance detection [4]. The MMN is generated, even in the absence of attention, when an infrequent (deviant) sound occurs among frequently repeated (standard) sounds, and peaks at 100–250 ms from the onset of sound change [4]. It is also elicited by an infrequent combination of sound attributes, called feature conjunction [5–7]. The MMN is characterized by a typical frontocentral scalp distribution, with positive voltages at electrodes below the Sylvian fissure, which is consistent with generators located bilaterally in the region of the secondary auditory cortex, including the superior temporal gyrus (STG) and the anterior Heschl’s gyrus (HG) [8–11]. Additional generator sources have been located to prefrontal cortex [12–14]. The MMN has been proposed as an objective tool for the evaluation of automatic central sound discrimination in a range of neurologic, psychiatric and neurodevelopmental conditions [15–17], and recently the protocols to obtain the MMN to multiple auditory contrasts simultaneously have been optimized (i.e., the so-called multi-feature paradigm [18–20]). In particular, there is growing promise to use the MMN as a break-through biomarker for predicting onset of psychosis [21,22].

However, recent research has shown that the MMN can no longer be considered the earliest correlate of auditory deviance detection in humans. Indeed, a range of recent studies have revealed that auditory deviations from a regular sound sequence are reflected by modulations of much earlier AEPs than the MMN, such as the middle latency response (MLR), peaking at latencies from 20 to 50 ms after stimulus onset [23–33]. The MLR is characterized by a sequence of waveforms in the latency range of 12–50 ms from sound onset, labeled N0, P0, Na, Pa, and Nb, which represent the earliest cortical response to a sound.

Also, early correlates of deviance detection can be seen in AEPs at the level of the brainstem, as shown by the frequency following response (FFR) [34]. The FFR follows the phasic brainstem response starting at 5-10ms after sound onset. It reflects the sustained evoked potential based on precisely phase-locked responses up to 1000 Hz of neuronal populations in the auditory brainstem of the ascending auditory pathway [35]. By means of measuring the FFR elicited to changes in speech syllables, Slabu et al. [34] demonstrated genuine novelty detection based on the subcortical encoding of auditory regularities. This finding was confirmed by a recent event-related fMRI study where a frequency oddball paradigm was applied, which revealed that true auditory deviance detection occured in the left inferior colliculus (IC) and in the bilateral medial geniculate body (MGB) [36].

All the findings highlighted so far are in agreement with extensive research in animals, using the technique of single unit recordings. This research has disclosed stimulus-specific adaptation (SSA) as one of the major neuronal mechanisms underlying regularity encoding and deviance detection along the auditory pathway [37–39]. Altogether, this line of research has proposed that deviance detection, based on regularity encoding [1,40] is a pervasive property of the whole auditory system, spanning from lower levels of the auditory pathway to higher-order regions of the auditory cortex [24,39,41]. This raises the question of whether early correlates of deviance detection, such as those obtained within the latency range of the MLR and even at the level of the brainstem with the FFR, could improve the diagnostic and prognostic value of the MMN in clinical studies. However, obtaining correlates of deviance detection in the early latency ranges is challenging because these AEPs show lower signal-to-noise ratio (SNR) compared to the MMN and are very small in amplitude, which is due to the size of anatomical areas involved [42], and therefore require tailored paradigms to be properly recorded. Previous attempts could report simultaneous recordings of the MMN and MLR deviance-related correlates [18,19,23,27], but to date, no study provided simultaneous recordings of deviance-related effects in the long-latency range (MMN), the MLR, and the FFR. Moreover, since the different levels of deviance detection along the auditory hierarchy might reflect different specific processes and are sensitive to different kind of regularities (i.e., simple to complex along the hierarchy [32]) finding a common recording protocol disclosing deviance responses along the auditory hierarchy may provide useful information regarding the organization of the auditory novelty system [39] and may result in a useful clinical tool. Therefore, the aim of the present study was to investigate whether subcortical and cortical deviance-related responses could be recorded with a single recording protocol along the whole auditory hierarchy.

## Materials and Methods

### Participants

Data were collected from 20 healthy participants (13 female, 7 male, aged 19–31 years, mean age ± standard deviation [SD] = 22.25 ± 3.29). All participants were tested for normal hearing and audiometric tests showed that each participant had a mean hearing threshold below 20 dB sound pressure level (between 250 and 3000 Hz). Additionally, they were asked to complete a health questionnaire in order to screen for any history of neurological or psychiatric disease. Two participants were excluded because their SNR in the FFR analysis was below 1.5, so that the final number of subjects used for analysis was 18 (11 female, 7 male, aged 19–31 years, mean age ± SD = 22.56 ± 3.33). All participants gave written informed consent and received compensation for participating in the study. The study was approved by the Ethical Committee of the University of Barcelona, and was conducted according to the Declaration of Helsinki.

### Stimuli and Procedure

Participants were seated in a comfortable chair in an electrically shielded and sound-attenuated room. They were asked to relax, concentrate on a silent movie with subtitles and to ignore the experimental sounds.

The auditory sequence was presented binaurally with a constant stimulus onset asynchrony (SOA) of 363 ms through ER-3A insert earphones (Etymotic Research, Elk Grove, IL, USA) with an intensity of 75 dB SPL. The stimuli were sine waves of 150 ms duration (including 5 ms rise and 5 ms fall time) with a carrier frequency of 2230 Hz. These sine waves were amplitude-modulated (AM) with a symmetric triangle function (modulation depth was 100%). Stimuli were presented in two different auditory conditions: the oddball and reversed-oddball conditions. In the oddball condition, the modulation frequency was 290 Hz for the standard stimuli and 410 Hz for the deviant stimuli. The deviant probability was set at 0.2, and a total of 1010 deviants were presented. In the reversed-oddball condition, the modulation frequency was 410 Hz for the standard stimuli and 290 Hz for the deviant stimuli, that is to say, standard and deviant stimuli from the oddball condition switched their roles. The presentation probabilities were the same as in the oddball condition. The total number of standard tones presented in the reversed-oddball condition was 1008. Note that this condition was implemented to allow for the comparison of brain responses elicited to the same physical sounds presented in the role of deviants (oddball) and in the role of standards (reversed-oddball). In other words, this condition was set to control for the physical characteristics of the stimuli, as it is very well established that early sensory auditory evoked responses depend on the physical features of the eliciting stimuli (i.e., modulation frequency in the present experiment; Picton et al. [43]). The oddball condition was presented in five blocks, with each block containing 1010 trials. The reversed-oddball condition was presented in two blocks, each containing 630 trials. Otherwise, conditions were intermixed randomly. Sound presentation was controlled with the software MATLAB (MathWorks, Natick, MA, USA), using the Psychophysics Toolbox extensions [44–46]. The total recording time was approximately 38 minutes plus breaks.

### Data acquisition

Electroencephalographic (EEG) signals were recorded with the Neuroscan 4.4 acquisition software (Compumedics NeuroScan, Charlotte, NC, USA) from 36 scalp electrodes mounted on an elastic nylon cap (Quik-Cap, Compumedics NeuroScan) according to the 10–20 system. An electrode placed on the right earlobe (A2) was used as online reference, and EEG data were grounded midway between Fz and FPz. In addition, an electrode was placed on the left earlobe (A1), each mastoid (M1, M2), and the tip of the nose. For recording eye movements, two bipolar electrodes were placed above and below the left eye (vertical electrooculogram) and at the outer canthi of each eye (horizontal electrooculogram). During the EEG recordings, all electrode impedances were kept below 10 kΩ. EEG signals were amplified using a SynAmps RT amplifier (Compumedics NeuroScan), band-pass filtered from 0.05 to 3000 Hz and digitized with a sampling rate of 20000 Hz.

### Data analysis

For the EEG analysis, the software MATLAB (MathWorks) and the toolbox EEGLAB [47] were used. The signal of broken electrodes was interpolated [48].

### Frequency Following Response

For FFR analysis, continuous data were filtered off-line with a Kaiser-windowed sinc band-pass filter from 120–1500 Hz and re-referenced to the averaged signal of all scalp electrodes (from each electrode for each time point). An adaptive filter was used to remove harmonics of 50 Hz power line noise (CleanLine toolbox for EEGLAB [49]) [50]. Epochs of 210 ms were used, including a baseline of -50 ms relative to stimulus onset. Epochs with any activity exceeding a range of ±35 μV were rejected. To analyze the responses in the frequency domain, fast Fourier transform (FFT) was applied on the EEG averages windowed with a Hanning window within the 10–140 ms time period. The mean power of the responses to deviants and reversed-standards was calculated at the electrode CPz for a 10 Hz wide bin surrounding 410 Hz (F_0_ of the modulation function). To calculate the SNR, the mean amplitude of the response to the modulation F_0_ (405–415 Hz) was divided by the mean amplitude of the noise level, which was defined as the 30 Hz bins from 370 to 400 Hz and 420 to 450 Hz. Two subjects whose SNR was smaller than 1.5 in both conditions were excluded from subsequent analysis. A paired t-test was conducted on the mean power of the FFRs recorded to reversed-standards and deviants.

### Middle Latency Response

For the MLR range, data were filtered using a band-pass finite impulse response (FIR) filter from 15 to 200 Hz and re-referenced to the averaged signal of all electrodes (from each electrode for each time point). Epochs of 150 ms were used, including a baseline of -50 ms relative to stimulus onset. Epochs with amplitudes larger than ±100 μV were excluded from further analysis. Additionally, independent component analysis (ICA) with the Second Order Blind Identification (SOBI) method [47, 51] was performed to remove artifacts. Epochs were averaged separately for reversed-standard and deviant stimuli. The grand average peak latencies were identified for each MLR component (Na, Pa and Nb). Individual mean amplitudes were extracted from an 8 ms window centered on the grand average peak latency of each component at F3, Fz and F4 electrodes. Accordingly, mean amplitudes were obtained for latencies between 20–28 ms (Na), 28–36 ms (Pb) and 38–46 ms (Nb). For each component, a 2 × 3 repeated measures analysis of variance (ANOVA) was conducted using the factors Stimulus Type (deviant, reversed-standard) and Electrode (F3, Fz, F4). Bonferroni corrections were applied to adjust *p*-values for testing on three MLR components. In addition to F- and *p*-values, the effect size (partial eta squared; η_p_^2^) was calculated for all repeated measures ANOVAs.

### Long Latency Response: Mismatch Negativity

For long latency response (LLR) analysis, data were filtered using a band-pass FIR filter from 0.2 to 30 Hz, and were re-referenced to the nose electrode. Epochs of 500 ms were used, which included a -100 ms baseline relative to stimulus onset. Epochs with amplitudes larger than ±100 μV were excluded from further analysis. Epochs were averaged separately for reversed-standard and deviant stimuli. A 40-ms window around the grand-average peak latency of the difference wave was used to calculate individual mean amplitudes elicited at the electrodes F3, Fz and F4. This window ranged between 100 and 140 ms. A 2 × 3 repeated measures ANOVA was conducted on the mean amplitudes extracted from the LLR time window with the factors Stimulus Type (deviant, reversed-standard) and Electrode (F3, Fz, F4). In addition to F- and *p*-values, the effect size (partial eta squared; η_p_^2^) was calculated.

## Results

### Frequency Following Response

**Fig 1:**
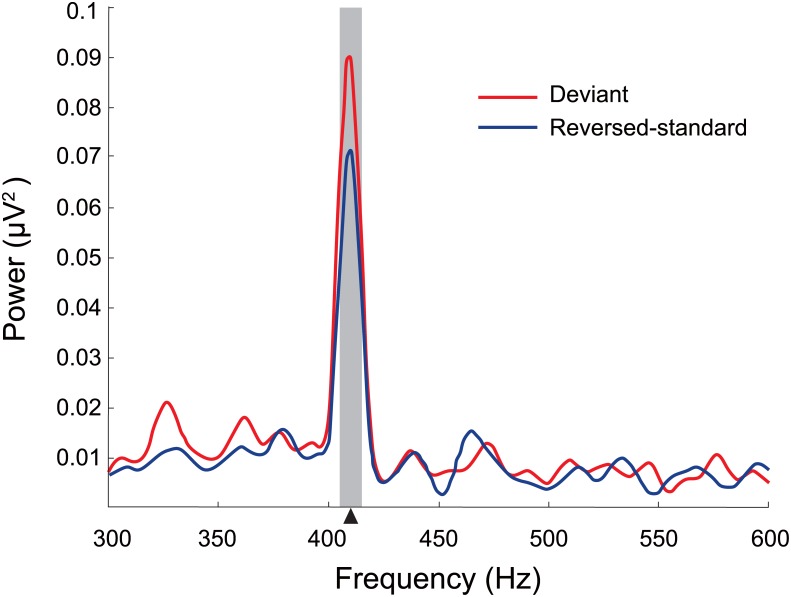
Grand average power spectrum of FFR at CPz electrode yielding a maximum peak at the modulation frequency (410 Hz). The waveforms are shown for reversed-standard (blue) and deviant (red) stimuli. The grey shaded bar denotes the window of the mean power used for statistics (405–415 Hz). The arrow on the x-axis indicates the modulation frequency (410 Hz). Note the significant enhancement of the spectral power at 410 Hz elicited by deviant stimuli (*p* < 0.05).

Fig 1 shows the grand average power spectrum of the FFR at CPz electrode. The mean power within 405–415 Hz was 0.075 μV^2^ (SD = 0.076, standard error [SE] = 0.018) for deviant and 0.058 μV^2^ (SD = 0.076, SE = 0.017) for reversed-standard stimuli. A paired t-test showed that the power of the FFR to deviant stimuli at CPz was significantly greater than that observed in response to reversed-standard stimuli (*t*[17] = 2.39, *p* < 0.05).

### Middle Latency Response

**Fig 2:**
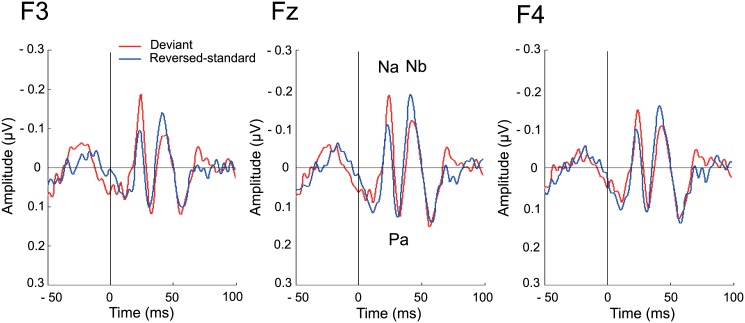
Middle-latency responses at the F3, Fz, and F4 electrodes. MLR waveforms are shown in response to reversed-standard (blue) and deviant (red) stimuli. The Na component was significantly enhanced by the deviant stimuli (corrected p < 0.005). Also, a significant attenuation to the deviant compared to reversed-standard stimuli was observed at the Nb component (corrected p < 0.005).

Fig 2 shows the grand average of the MLR at F3, Fz and F4, and the mean amplitudes at each component are described in Table 1. For the Na component (20–28 ms), a significant main effect of Stimulus Type was observed, with larger amplitudes elicited by the deviants compared to reversed-standards (*F*[1,51] = 13.007, *corrected p* < 0.005, η_p_^2^ = 0.203), but there was no main effect of Electrode (*F*[2,51] = 0.051, *corrected p* = 1.000, η_p_^2^ = 0.002). No Stimulus Type × Electrode interaction was observed (*F*[2,51] = 0.115, *corrected p* = 1.000, η_p_^2^ = 0.004) either. Analysis of the Pa component (28–36 ms) yielded no main effect of Stimulus Type (*F*[1,51] = 0.643, *corrected p* = 1.000, η_p_^2^ = 0.004), Electrode (*F*[2,51] = 0.114, *corrected p* = 1.000, η_p_^2^ = 0.004), or Stimulus Type × Electrode interaction (F[2,51] = 0.124, *corrected p* = 1.000, η_p_^2^ = 0.005). For the Nb (38–46 ms) component, a significant main effect of Stimulus Type was observed with smaller amplitudes elicited by the deviants compared to the reversed-standards (*F*[1,51] = 15.230, *corrected p* < 0.005, η_p_^2^ = 0.230). No main effect of Electrode (*F*[2,51] = 0.341, *corrected p* = 1.000, η_p_^2^ = 0.013) and no significant Stimulus Type × Electrode interaction (*F*[2,51] = 0.104, *corrected p* = 1.000, η_p_^2^ = 0.004) were observed.

**Table 1:** Mean and Standard Error (in parentheses) of MLR component amplitudes (in microvolts) at Fz.

	Na		Pa		Nb	
Deviant	-0.115	(0.025)	0.075	(0.03)	-0.095	(0.033)
Reversed-standard	-0.058	(0.028)	0.079	(0.021)	-0.15	(0.035)
Difference	-0.056	(0.018)	-0.004	(0.021)	0.055	(0.02)

**Fig 3:**
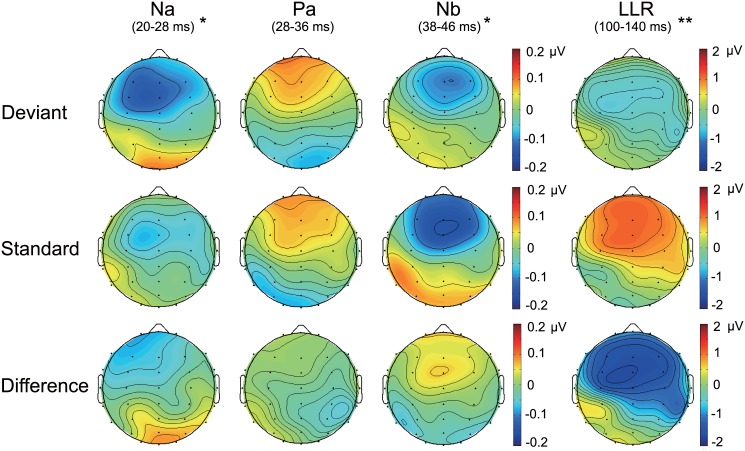
Scalp topographies for MLR and LLR. Topographic maps are shown for middle-latency (Na, Pa, and Nb component) and long-latency responses to deviant and reversed-standard stimuli, and their corresponding difference waveform (deviant—reversed-standard). * *p* < 0.01, ** *p* < 0.001

Fig 3 shows the topographic maps of the Na (20–28 ms), Pa (28–36 ms), and Nb (38–46 ms) components. The Na elicited to reversed-standard and deviant stimuli was distributed over the left frontocentral area, but the repeated measures ANOVA revealed no significant main effect of Electrode as stated above.

### Long Latency Response: Mismatch Negativity

**Fig 4:**
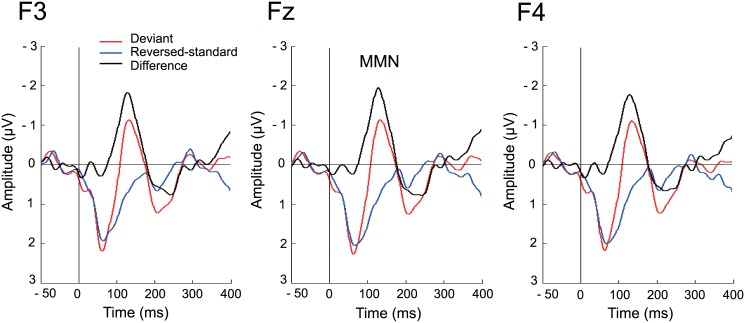
Deviance-related long-latency responses at the electrodes F3, Fz, and F4. Waveforms for reversed-standard (blue) and deviant (red) stimuli as well as their corresponding difference waveforms (deviant—reversed-standard; black) are shown. The MMN peaks at approximately 120 ms after stimulus onset, yielding a significant difference between the reversed-standard and deviant stimuli (*p* < 0.001).

Fig 4 shows the grand average of the LLR at F3, Fz and F4. MMN peaked in the grand average at about 120 ms after stimulus onset. The mean amplitude of MMN in the latency range of 100–140 ms was -1.579 μV (SD = 1.482, SE = 0.349). The repeated measures ANOVA showed a significant main effect of Stimulus Type, (*F*[1,51] = 62.085, *p* < 0.001, η_p_^2^ = 0.549), but no main effect of Electrode (*F*[2,51] = 0.053, *p* = 0.949, η_p_^2^ = 0.002). There was no significant Stimulus Type × Electrode interaction either (*F*[2,51] = 0.037, *p* = 0.964, η_p_^2^ = 0.001).

The topographic maps of the LLR within 100–140 ms from stimulus onset are shown in Fig 3. There was a trend towards the MMN being preferentially distributed over the left hemisphere, but no significant main effect of Electrode was observed as shown above.

## Discussion

The results obtained in the present experiment highlight that auditory deviance detection is a property of the whole auditory hierarchy. Indeed, the amplitude enhancements observed at different latency ranges, namely FFR, MLR and LLR, support this recent view of a pervasive auditory novelty system [39,41]. The amplitudes of the difference signal increased as it engaged higher order processing, which is due to the increasing extent of the brain areas involved in each of the successive responses [8–11, 42]. In particular, our electrophysiological results revealed that the spectral power of the FFR at the modulating frequency (i.e., 410 Hz) was enhanced when the stimulus occurred with a low probability in the role of a deviant, compared to its spectral power when presented frequently as reversed-standard. Moreover, the results revealed a larger amplitude of the Na component of the MLR elicited by deviant stimuli, at about 20-28ms from stimulus onset, as well as an attenuation of the Nb component elicited by deviant stimuli at about 38-46ms. We also observed a typical MMN elicited to the deviant stimuli with a peak latency of about 120 ms. Remarkably, these effects were obtained simultaneously within the same sample of participants and with one single recording protocol.

We were able to demonstrate that AM deviant stimuli modulated the FFR of the auditory brainstem. AM involves changing the amplitude of a carrier wave using another modulator wave. The frequency of the carrier signal remains unchanged but its amplitude is varied in accordance with the amplitude of the input signal. Therefore, AM is more complex sound than pure tones and thought to be important in speech intelligibility [52,53]. Neural activity in the auditory brainstem is synchronized to the modulation frequency, which can be observed in the FFR. Several animal studies have shown that the anatomical level of the brainstem at which neurons are synchronized to AM sounds depend in fact on the modulation frequency [53]. In particular, frequencies of circa 400 Hz appear to entrain subcortical stations of the auditory pathway including the auditory brainstem [53]. Therefore, the results of the present experiment parallel previous results from our laboratory obtained in humans that revealed true deviance detection based on regularity encoding in the IC by using consonant-vowel syllables as stimuli [34] or the use of wide-band white noise bursts [36], and are in agreement with animal studies showing SSA in the IC (see Escera & Malmierca [39]). However, while no deviance-related responses in the long latency range (i.e., MMN) were reported in the study by Slabu et al. [34], the present protocol allowed us to obtain, with one single recording protocol, these two correlates of deviance detection in humans concurrently.

Regarding the MLR, the results of the present study revealed that AM frequency deviants modulated brain responses at the latency range of the Na component. Previous studies had shown that the Na component was modulated by location deviants [24–26]. Additionally, Althen et al. [31] showed that the Na-Pa complex was modulated when presenting intensity deviants. The results obtained here are the first to demonstrate that in addition to location and intensity, manipulations in modulating amplitude of a carrier frequency can trigger deviance-related responses by the Na component of the MLR. Previous studies observed component-specific effects for particular feature changes, such as on the Nb for frequency changes [23,28,30,32,33,54], on Na for location changes [24–26] as stated above, and on the Na-Pa transition for intensity changes [31]. It was hence proposed that this component specificity would in fact reflect the encoding of the corresponding physical features of acoustic stimuli by the generating neural populations of the corresponding components [41], yet the present results challenges in fact, this theoretical proposal.

Interestingly, while the deviant stimuli increased the amplitude of the Na response of the MLR, we also observed an attenuation of the Nb component’s amplitude. Our findings are in contrast with previous studies that found an enhancement of the Nb component to frequency deviants. Grimm et al. [23] reported evidence that the enhancement of Nb amplitudes in response to frequency deviants reflected a genuine deviance-detection, which was corroborated by Recasens et al. [30]. In a multi-feature paradigm in which standards and several different other types of deviant stimuli were presented alternately (see details in Näätänen et al. [18]), frequency deviants elicited an enhanced Nb response [28]. By comparing MLRs in a frequency oddball condition and a feature-conjunction condition (with both frequency and location changed in the deviants), Althen et al. [32] demonstrated that the Nb component was enhanced by deviants in the oddball condition, whereas no such difference was observed in the feature-conjunction condition. The discrepancy between our findings and previous studies might be due to the fact that we used AM pure tones. The difference of the acoustic features of these sounds may have affected how deviance, and in fact the stimulus features themselves, are encoded in the brain.

A more speculative, alternative interpretation can be put forward for the Na enhancement/Nb attenuation found in the present experiment. Broadly speaking, the EEG signal arises from synchronized post-synaptic potentials of a particular set of cortical pyramidal neurons. The polarity of the EEG signal measured at the scalp electrodes depends on the particular orientation of the generating dipoles. Although pyramidal cells are oriented perpendicular to the surface of the cerebral cortex, their orientation relative to the scalp is not specific because of the presence of gyri and sulci in the cerebral cortex. Since cortical sulci would produce tangential dipoles and cortical gyri would produce radial dipoles [55], if deviant stimuli stimulate a particular neural population which had a different orientation than that responding to the reversed-standard in the same latency range, then it is possible that the deviants would yield an attenuated response. According to Yvert’s intracranial AEPs study [42], P0 and Na were generated in the postero-medial part of HG or Heschl’s sulcus (HS), and Pa/Pb were generated over the supratemporal plane successively involving the first Heschl’s gyrus (H1)/HS, the planum temporale, H2/H3 when present, and the STG. Thus, MLRs arise from a number of gyri and sulci in auditory cortex. Moreover, recent magnetoencephalographic studies have revealed that source generators of the transient Nbm component have been located over lateral areas of the STG [56], whereas the deviance-related Nbm for frequency change has been located over the anterior rim of HG [30], suggesting that the deviance-related Nb component may arise from a different cortical area from that responding to reversed-standards. Based on these findings, AM frequency deviants, stimulating different cerebral region from that responding to reversed-standards, might generate post-synaptic potentials of cortical pyramidal neurons whose dipoles were opposed to the reversed-standards, and then attenuate, rather than enhance, the Nb component at the scalp electrodes. This notion may also explain the diverged transitions between each MLR component as shown in Fig 2. Interestingly, Althen et al. [31] also showed a deviance-related divergence at the transition from the Na to Pa component in response to an intensity change of click sounds.

According to the topography of MLR and MMN elicited by pure tones in previous studies, the MMN to frequency, intensity and duration change showed a right-side distribution [57], whereas the Nb component to a frequency change showed a central distribution [32]. The MMN and Na components elicited to AM frequency changes in the present topographic maps showed a left-side distribution, although no significant difference of amplitudes between the electrodes was observed. The results were not in accordance with those previous studies, implying that the AM sounds might facilitate another generator in each response. On the other hand, there was no significant Stimulus Type × Electrode interaction for MMN analysis in this study. That is, the evoked responses to standard and reversed-standard stimuli were similar in distribution, suggesting that the change in amplitude (i.e., main effect of stimulus type) was driven primarily by neural adaptation. Indeed, neural adaptation was thought to be representative neuronal mechanism for auditory sensory memory reflected by MMN [37,58,59]

Taken together, our findings provide further evidence that deviance detection based on regularity encoding is a pervasive property of the auditory system. Importantly, we were able to demonstrate, in a single recording protocol, that novelty responses occur at multiple levels of the auditory hierarchy. Direct evidence has come from a separate line of research performed in single-unit recordings in animals. It is known that a particular class of neurons show a decreased firing rate when repetitive frequent sounds are presented in an oddball paradigm, a phenomenon termed SSA and increase their responses when novel sounds are presented. Such neurons are often termed novelty neurons. Novelty neurons have been found not only in primary auditory cortex [60–62] but also in auditory subcortical stations such as MGB [63,64] and IC [65,66]. Corresponding to these neural responses, genuine novelty-related electrophysiological responses were found in the human auditory brainstem as shown by the FFR as well as in the auditory cortex in the MLR and LLR. Although the standard and reversed-standard stimuli were the same physical sounds in the present study, a proper control condition taking into account refractoriness was not applied (see Schöger & Wolff [67]). Therefore, the MMN, the enhanced FFR and the enhanced Na responses to AM frequency deviants may imply that the standard responses in the oddball blocks result from neural adaptation, and the reversed-standard responses in the reversed-oddball blocks may represent the restoration of the neural adaptation. Our study demonstrated that deviance detection responses can be recorded in humans in various anatomical regions and latency ranges with a single recording protocol. The early different responses revealed in the MLR imply that the pitch difference was quickly detected (within the first few milliseconds). In contrast, the FFRs are the responses based on the whole duration of the sound. Therefore, FFR and MLR may have different temporal integration functions which might affect the deviance-related responses in each anatomical level.

Recently, Bidelman [68] presented a new stimulus paradigm to record FFR and cortical AEPs in the LLR range to speech syllables. Additionally, combined scalp-recorded subcortical and cortical perceptual processes have been reported based on auditory encoding [69–71]. In the present study, we went a step further by creating a paradigm that allowed us to record the MLR in addition to the FFR and the LLR when manipulating the AM frequency and keeping the carrier frequency constant. Furthermore, with the present design we could examine not only brain responses to the encoding of auditory stimuli (i.e., to the standards and reversed-standards) but also deviance-related responses at different levels of the auditory hierarchy. Optimizing the recording of both subcortical and cortical responses is of paramount importance in reducing the time needed to assess cognitive auditory function so that it can be implemented in clinical settings [59]. Some psychiatric disorders such as schizophrenia and developmental disorders are known to show MMN deficiency [21,72,73], reflecting cognitive decline. By applying our paradigm in these patients, we might distinguish the pathophysiology of these disorders based on auditory discrimination processes, and develop it to help the diagnoses in the future.

## Conclusions

The MMN provides by the latency range of the LLR of the AEPs, a correlate of automatic auditory discrimination in human auditory cortex. Recently, however, deviance-related responses were observed in early processing stages by the latency range of the MLR components, and even at subcortical level as reflected by the FFR. In the present study, we showed that AM frequency deviants modulate the FFR, the Na and Nb components of the MLR, and the LLR as shown by the elicitation of MMN, and that these modulations can be recorded concurrently with one single recording protocol. Thus, our findings support the notion that novelty detection is a pervasive property of the auditory system’s hierarchy and provide a new research and clinical tool to foster studies in cognitive and clinical auditory neuroscience.
